# Pre- and Postoperative Evaluation by Photoplethysmography in Patients Receiving Surgery for Lower-Limb Varicose Veins

**DOI:** 10.1155/2014/562782

**Published:** 2014-02-19

**Authors:** Orlando Adas Saliba Júnior, Mariangela Giannini, Ana Paula Mórbio, Orlando Saliba, Hamilton Almeida Rollo

**Affiliations:** ^1^Faculty of Medicine of Botucatu, Universidade Estadual Paulista (UNESP), Avenida Prof. Montenegro, Distrito de Rubião Junior, s/n, 18618970 Botucatu, SP, Brazil; ^2^Federal University of Mato Grosso, Campus of the Araguaia, Avenida Universitária No. 3.500, 78698-000 Pontal do Araguaia, MT, Brazil; ^3^Araçatuba Dental School, Universidade Estadual Paulista (UNESP), Rua José Bonifácio, 1193, Vila Mendonça, 16015-050 Araçatuba, SP, Brazil

## Abstract

*Objective*. To evaluate the effectiveness of surgery in treating primary varicose veins in the lower limbs by photoplethysmography (PPG) and duplex mapping (DM). *Method*. Forty-eight lower limbs were clinically evaluated according to the CEAP classification system and subjected to PPG and DM exams. Each limb had a venous refill time (VRT) of <20 seconds and a normal deep vein system (DVS) by DM. *Results*. The mean pre- and postoperative VRTs were 13.79 and 26.43 seconds, respectively (*P* < 0.0001). After surgery, 42 limbs (87.50%) had normal results by PPG (VRT > 20 seconds). Four limbs (8.33%) showed improved VRTs, but the VRTs did not reach 20 seconds. In the 2 limbs (4.17%) that maintained their original VRTs, the DM exams showed the presence of insufficient perforating veins. *Conclusion*. In most cases, PPG allows for a satisfactory evaluation of the outcome of varicose vein surgery.

## 1. Introduction

Most vascular surgical centres use a clinical exam to evaluate patients undergoing surgery for varicose veins. When carefully performed, this exam always diagnoses chronic venous insufficiency (CVI). Some clinical classifications for venous insufficiency (VI) have been described, but most do not allow for a complete and satisfactory clinical analysis of the disease [1]. To ensure uniformity in the diagnostic and evaluation records for different modes of treatment, the Society for Vascular Surgery and the International Society for Cardiovascular Surgery have proposed the CEAP classification for universal use, which covers the Clinical, Etiological, Anatomical, and Physiopathological aspects. The clinical component of this classification contains categories that are arranged in increasing order of severity [2–4]. However, in individuals with VI, a clinical evaluation of the limbs does not identify the systems involved or their anatomical levels [5, 6], and it cannot provide sufficient and fundamental information for choosing a surgical technique [7]. Moreover, the clinical exam can be subjective and may not allow a quantitative evaluation of the surgical result.

As complementary methods, invasive and noninvasive diagnostic methods have been developed to evaluate venous function in patients with VI [2, 7–10]. Invasive tests, such as phlebography and direct measurement of the venous pressure, provide crucial anatomical and functional data that are needed to perform a safe surgical procedure [7]. However, because of their invasive character, these procedures cannot be continually repeated. Moreover, patients' acceptance of these tests is normally low, making them impractical as monitoring techniques [11].

Noninvasive methods are commonly used to diagnose and evaluate the effectiveness of different arterial and venous diseases [12–17]. The most widely used noninvasive methods are photoplethysmography (PPG), air plethysmography (APG), and duplex mapping (DM). These tests are more economical and cause less discomfort to patients compared to invasive methods [18, 19]. DM is considered the gold standard among the noninvasive methods [20] for venous diseases of the lower limbs because it yields quantitative and qualitative evaluations. However, DM is very expensive to perform. The introduction of a protocol for using PPG to evaluate patients in the preoperative period for varicose vein surgery reduced the number of surgeries at a vascular clinic from 30% to 24% [21].

As an easily performed exam that does not require the operator to have lengthy training, PPG allows venous reflux to be identified and quantified [22]. PPG can be very useful in the postoperative evaluation of patients who have undergone primary vein surgery to correct reflux. The results obtained by PPG can be correlated with results of the direct measurement of the venous pressure and phlebography [6, 8, 22, 23]. Various studies have used PPG to evaluate the results of varicose vein surgery or CVI demonstrating its usefulness [24–27]. PPG has also been used to evaluate the effects of venotonic drugs before and after treatment in patients with vein diseases allowing differences between groups to be verified [28].

Some authors have observed differences between the results of PPG and clinical findings [28, 29]. Some studies verified the association between the PPG results, CEAP clinical evaluation, and venous refill time (VRT) and found a good correlation with duplex ultrasound in 246 patients treated with foam sclerotherapy. In a study of 28 people who were divided into groups based on the presence and seriousness of venous reflux, PPG was able to distinguish normal limbs from limbs with reflux, although it could not gauge the seriousness of the reflux [7]. PPG has been compared to APG and DM [19, 30]. Bays et al. [19] used APG, PPG, and DM to evaluate serious VI. The VRT sensitivity of PPG in identifying reflux was 100%, but its specificity was only 60%. The correlation between DM and APG was 0.83, although the coefficient of correlation between DM and PPG was 0.47. Evangelista and Fonseca [27] observed 36 limbs in 20 patients who had lower-limb varicose veins, reporting a sensitivity of 65.5%, specificity of 85.7%, and accuracy of 64.9% when comparing PPG with DM. In a study tracking patients at high risk for deep vein disease, PPG yielded results of 100% sensitivity and 73.8% specificity. Compared to ultrasonography, PPG was considered the best and most useful method for reaching this goal [31]. The reproducibility of PPG has been tested with patients in seated and standing positions, after standard exercise, with good results being observed [32].

Given the importance of the clinical evaluation and the usefulness of PPG, the objective of this research was to compare the results of the clinical and PPG examinations of patients undergoing surgery for primary varicose veins of the lower limbs. Evaluations were performed before and after surgery, and the results of the surgeries were evaluated.

## 2. Materials and Methods

This study included 40 male and female patients with primary varicose veins of the lower limbs who were seen at the Vascular Surgery Unit of the Clinical Hospital of the Botucatu Faculty of Medicine of the State University of São Paulo (Universidade Estadual Paulista (UNESP)), Brazil. Because some patients had changes in both lower limbs, a total of 48 limbs were analyzed. The inclusion criteria were as follows: written consent; clinically evaluated varicose veins, with a history suggesting that they were primary; VRT < 20 seconds in the PPG exam; and a normal DVS on the DM. The Research Ethics Committee of the Botucatu Faculty of Medicine, UNESP, approved the research. The study protocols were consistent with all regulations regarding research involving human subjects. All included research patients provided their written informed consent before participation.

For patient evaluation, the CEAP clinical classification system was used [2, 4]. The members were classified according to the most severe clinical signs; however, they could present some or all of the signs associated with the lower classes. After clinical evaluation, as a preoperative routine, all patients received PPG (Medacord PVL Enhanced Photoplethysmograph, Medasonics Inc.) and DM exams (Platinum machine, Philips-Color Velocity Imaging). An environment with stable light and temperature was employed, because luminosity variations could lead to changes in the PPG results, and temperature variations could lead to changes in VRT because of vasomotor changes in the skin's circulation. The DM exam was performed to verify that the DVS was normal and to examine changes to the superficial venous system (SVS) and the perforating veins. At 30 and 180 days after the operation, the patients again underwent PPG and clinical exams to evaluate the results of the surgery. If a patient's VRT value was not ≥20 seconds, then the DM exam was repeated.

**Figure 1:**
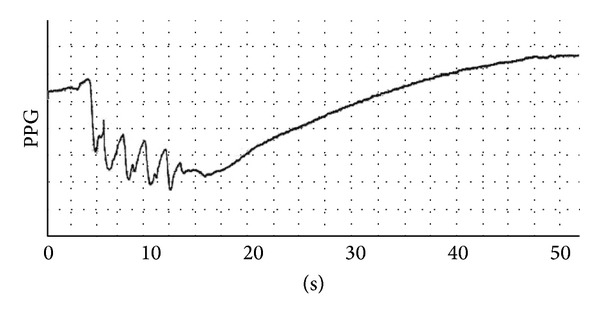
PPG graph with normal values. VRT = 32 s, VC1 = 13 s, and VC2 = 45 s.

The PPG exam was performed according to the technique described by Barnes et al. [33] and Nicolaides and Miles [23]. The patient was seated on a stretcher with his/her legs dangling. The PPG probe or sensor was attached to the skin in the medial area, in the distal third of the leg (the supramalleolar area) [22]. In the first step of the exam, the sensor was positioned and the patient was asked to make 5 movements to flex and extend the foot, leaving one leg in a relaxed position. After these movements, the machine traced a graph that, when stabilized, meant the end of the filling of the leg's venous bed. In the second step, with the sensor in the same position, a tourniquet with automatic inflation was placed above the knee. The tourniquet was attached to the machine and inflated with a predefined pressure of 45 mmHg, which is sufficient to block SVS [34]. Finally, the same movements as in step 1 were performed, the tourniquet was moved to below the knee, and the same sequence of movements as above was repeated. The Figure 1 shows a PPG graph with normal values.

The exam with the tourniquet on leg and thigh was repeated in 41 of the 48 limbs. The VRT was measured indirectly through the venous return and through the valve incompetence, based on a curve departing from a baseline, for each patient. An increase in VRT of >3 seconds when using the tourniquet was considered an improvement in VRT.

The data were subjected to statistical analyses by nonparametric tests with a 5% significance level, considering the nature of the variables and the population size of the study. Wilcoxon's test was used to compare the results from the PPG exams in the pre- and postoperative periods. The Friedman test was used to compare more than two related samples.

## 3. Results

This study included 12 male and 28 female patients (age range, 21–66 years). We examined 23 left limbs (48%) and 25 right limbs (52%) and performed 32 greater vein saphenectomies, 1 lesser vein saphenectomy, 10 preservations of the greater saphenous vein (double ligation of the saphenofemoral junction (SFJ)), 1 greater and lesser vein saphenectomy, 3 ligations of the perforating vein, and 1 tributary removal.

**Figure 2:**
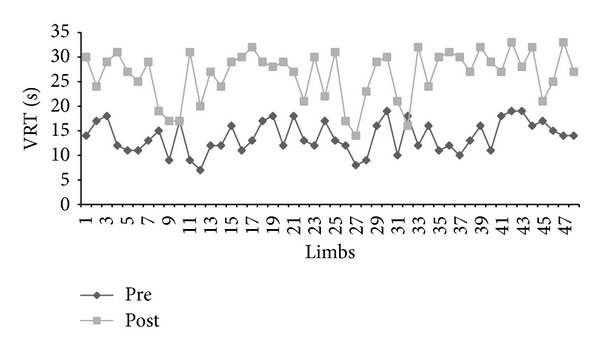
Venous refill time (VRT) by PPG in 48 lower limbs in the pre- and 30-day postoperative periods without tourniquet.

Figure 2 shows the results of the PPG exams, comparing the VRT results in the pre- and postoperative periods without tourniquet use. In 42 of the 48 limbs examined, the postoperative VRT was >20 seconds. Four limbs showed an improvement in VRT, but the VRT did not reach 20 seconds. Two limbs showed no postoperative improvement in the VRT. The average VRT values were 13.79 and 26.43 seconds in the pre- and postoperative periods, respectively. Most of the surgeries (87.5%) were regarded as satisfactory, according to the tests performed.

**Figure 3:**
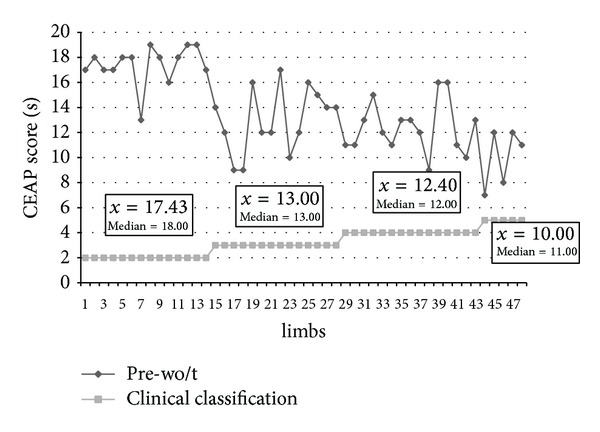
Clinical classification scores by CEAP and VRT results obtained by PPG of 48 lower limbs in the preoperative period without a tourniquet (Botucatu CH-FM, 2012).

In the preoperative period, the average VRT values in limbs with CEAP classification scores of 2, 3, 4, and 5 were 17.43, 13.00, 12.40, and 10.00, respectively. The median values for limbs from the same classes were very close to the averages (Figure 3). Comparisons of the results of the PPG exams between the CEAP clinical classes revealed differences between the results for class 2 and class 3 (*P* < 0.01), class 2 and class 4 (*P* < 0.001), and class 2 and class 5 (*P* < 0.001), but no differences between classes 3, 4, and 5 (*P* > 0.05). Based on the clinical exams, at 30 days after the surgery, most patients had no complaints, oedema, or residual varicose trajectories.

Table 1 shows the observed values and the calculated descriptive statistical parameters for the 41 limbs examined with and without a tourniquet. The average VRT for the 41 limbs in the preoperative period was 13.37 seconds without a tourniquet, 21.63 seconds with a tourniquet on the thigh, and 20.00 seconds with a tourniquet on the leg. In the postoperative period, the average VRT for the 41 limbs was 26.10 seconds. Comparison of the PPG results in the pre- and postoperative periods by Wilcoxon's test revealed a statistically significant difference (Table 2). Use of a tourniquet on the thigh or leg influenced the exam results.

The DM exam in the postoperative period showed the presence of insufficient perforating veins in the two limbs (4.17%) that did not show an improvement in VRT. For the 4 cases (8.33%) that displayed an improvement in VRT but did not reach a VRT of 20 seconds, we observed the presence of insufficient perforating veins in 1 case. In another case, it was observed rechanneling of the saphenous greater vein. This patient was previously submitted to saphenous greater vein preservation surgery with ligature of the SFJ. The other 2 limbs were from a patient with bilateral varicose veins, which were evaluated by PPG at 1 and 6 postoperative months. The changes observed at 1 month remained present at 6 months.

The descriptive statistical parameters indicated that there was no difference between the postoperative results at 30 and 180 days (*P* = 0.4332). Patients who had a normalized VRT at 1 postoperative month maintained a normal VRT on the PPG evaluation at 6 months. The 4 patients who had an improvement in VRT at 30 days but who did not attain a VRT of 20 seconds maintained their VRT values at 180 days. One of the 2 patients who did not have an improvement in VRT at 1 month had a normalized VRT at 6 months.

## 4. Discussion

In this study, we verified the usefulness of the PPG exam and its relationship to the clinical findings and the DM exam results for evaluating the results of surgery for lower-limb varicose veins. The VRT results showed a significant increase in the postoperative period in most of the limbs analyzed.

For a patient with varicose veins, a thorough clinical exam is fundamental for establishing a diagnosis, designing an adequate therapy, and obtaining a satisfactory prognosis. However, in many situations, both changes to the veins and the effectiveness of surgical interventions need to be evaluated [13]. In addition to not providing all of the necessary information, a clinical exam is subjective and, therefore, subject to variations. In this study, we used the CEAP clinical classification system [2–4] that is widely used in research and clinical practice [35–39]. Our goal in using this classification scheme was to standardize the exam method and facilitate comparisons.

In the preoperative clinical evaluation, the limbs had CEAP scores ranging from 2 to 5, with none from classes 1 and 6. Class 1, corresponding to telangiectasia or reticular veins, is generally indicated for treatment with sclerotherapy and/or microincision surgery. With many class 6 cases, it is not possible to perform PPG exams because of the presence of active ulcerated lesions on the legs. However, a study by Guillot [40] used PPG to evaluate coetaneous microcirculation in 19 patients with leg ulcers, with the author concluding that the results obtained by this method were compatible with those of other techniques.

We anticipated the inclusion of a greater number of female patients because primary varicose veins of the lower limbs affect women more than men [41, 42]. We used PPG in this study because it is a noninvasive method that has advantages over DM, including lower cost, ease of execution, and less training time for the examiner. PPG has shown a strong correlation with ambulatory venous pressure [8] and allows for the evaluation of reflux, with a satisfactory level of precision [13]. Although PPG does not have a high specificity when compared with imaging exams and is less precise in locating areas of insufficiency, it allows the disease severity to be evaluated and the effects of treatment to be predicted [43].

We considered VRT, a quantitative parameter obtained in the PPG exam, to be normal when it was ≥20 seconds, consistent with most published studies [12, 19, 25, 28, 44, 45]. To evaluate a method of effective measurement of the muscular force of the calf, Tucker et al. [46] considered 18 seconds to be a normal VRT. These authors repeated the exam with the application of a cuff above the knee when the VRT was <18 seconds, to evaluate the involvement of SVS and DVS. Sarin et al. [13] also considered a VRT ≤ 15 seconds to be abnormal.

In the preoperative period and as a complement to VRT evaluation, we used a tourniquet on the thigh and on the leg of 41 limbs, to analyze the contribution of the SVS in reflux. Comparisons between the exams without a tourniquet and with a tourniquet on the thigh and on the leg yielded statistically significant differences. Ten limbs showed an improvement in VRT values (increase ≥ 3 seconds) when the exam was performed with a cuff on the thigh compared to the exam without a cuff. Six limbs showed an improvement with a cuff on the leg, and 22 limbs had an improvement in VRT in both exams (with the cuff on the thigh and on the leg) when compared to the exam without a cuff. Only 3 patients did not have an improvement in VRT, even with the use of a cuff, although they showed an improvement in VRT in the postoperative period, with VRT > 20 seconds. These findings prove that, in most limbs, placing a cuff can predict the results of surgical treatment.

Iafrati et al. [47] considered that a VRT < 25 seconds indicated the use of a tourniquet to evaluate the contribution of the SVS. Complete VRT normalization after tourniquet placement indicates a diagnosis of superficial VI alone. If there is no improvement observed after compression with a tourniquet, then the diagnosis is simply deep VI. An increase of 5 seconds in VRT without complete normalization is indicative of deep VI with a component of superficial VI. In a study by Gaitini et al. [44] placing a cuff on normal limbs or on those with CVI did not change the VRT, but it prolonged VRT in limbs with primary varicose veins. In a study involving 1583 limbs, Cheng and Wong [43] observed a significant increase in VRT after applying a cuff to patients who had serious reflux in the SVS.

Another important finding of this work was the relationship observed between the CEAP clinical classification and the VRT obtained by PPG. Both exams determined the same tendency: when the clinical exam indicated a more serious clinical picture, the VRT results by PPG had lower values, indicating poorer conditions. The statistical tests confirmed that the PPG exam was able to differentiate a slighter grade from the others but did not differentiate the intermediate grades. In a study of 74 limbs comparing the results of CEAP, APG, PPG, and DM, Iafrati et al. [47] observed that only PPG and DM showed significant differences between normal limbs and those with CVI. According to the authors, no test discriminated stages 2 and 3 of the clinical classification system.

Thirty days after the operation, only 6 of the 48 limbs did not achieve a VRT ≥ 20 seconds, although 4 (8.33%) of these limbs had improved, considering that the VRT had an increase of >3 seconds. In addition, 42 limbs (87.50%) showed results considered normal in PPG. Thus, PPG showed that surgery achieved good results in most of the limbs evaluated.

In 2 of the 4 limbs in which the PPG exam showed an improvement in VRT values in the postoperative period, but which did not attain a value that this study considered normal, we performed DM. One of these limbs had GSV insufficiency, with reflux in the SFJ and trunk, which was corrected with a double ligation of the SFJ and GSV preservation. In this limb, the DM exam revealed that the SFJ ligation was open, with reflux newly occurring. Therefore, the still nonnormalized PPG indicated a suspicion that there might have been some change, which was identified by DM. In the other patient in which the VRT improved without attaining 20 seconds and in 2 patients in which the PPG did not show an improvement in VRT, DM found insufficient perforating veins in the leg, which were probably not ligated during surgery. Both patients showed improvement of clinical signs. However, because the PPG might not have been sensitive enough to detect low levels of VI, we cannot draw conclusions about these findings. New studies, involving a greater number of patients and related diagnostic methods, should be performed.

There was no change in the PPG results at 6 months when compared with those 1 month after the operation. This finding may suggest that a repetition of the PPG is not needed at 6 months unless the PPG showed changed values in the first month or if the patient's clinical state worsened during postoperative tracking.

## 5. Conclusion

The data presented in this study suggest that most of the surgeries performed for lower-limb varicose veins gave satisfactory results, based on the PPG and DM exam findings. The evaluated patients displayed differences in their pre- and postoperative VRT results. The PPG results correlated with the clinical findings, allowing for an evaluation of the results of surgeries for varicose veins of the lower limbs.

## Figures and Tables

**Table 1:** Venous refill times (seconds) for 41 lower limbs in the pre- and postoperative periods, with and without tourniquet use (Botucatu CH-FM, SP, 2012).

Period	Preoperative			Postoperative
Description	No tourniquet	Thigh tourniquet	Leg tourniquet	No tourniquet
Number of limbs	41	41	41	41
Arithmetic average	13.37	21.63	20.00	26.10
Standard deviation	3.22	5.84	7.01	5.09
Median	13.00	23.00	22.00	28.00
Minimum value	7.00	10.00	9.00	14.00
Maximum value	19.00	32.00	32.00	32.00
Normal distribution	No	Yes	Yes	No
Confidence interval	12.35–14.38	19.79–23.48	17.79–22.21	24.49–27.70

**Table 2:** Results of descriptive statistical tests (CH-Botucatu FM, SP, 2012).

Groups compared	Test	Value	Probability	Significance
Pre-wo/t × pre-t/t × pre-g/p	Friedman	38.413	<0.0001	Significant
Pre-wo/t × pre-t/t	Dunn's*	−47.000	<0.001	Significant
Pre-wo/t × pre-t/l	Dunn's*	−47.500	<0.001	Significant
Pre-t/t × pre-t/l	Dunn's*	0.500	>0.050	Not significant
Post-wo/t × post-wo/t	Wilcoxon	−818.000	<0.0001	Significant
Post-t/t × post-wo/t	Wilcoxon	−457.000	0.0015	Significant
Pre-t/l × post-wo/t	Wilcoxon	−566.000	0.0003	Significant

*Dunn's multiple comparison. Pre-wo/t: preoperative without tourniquet; pre-t/t: preoperative with tourniquet on thigh; pre-t/l: preoperative with tourniquet on leg; post-wo/t: postoperative without tourniquet.
